# SaQuant: a real-time PCR assay for quantitative assessment of *Staphylococcus aureus*

**DOI:** 10.1186/s12866-021-02247-6

**Published:** 2021-06-08

**Authors:** Colin Wood, Jason Sahl, Sara Maltinsky, Briana Coyne, Benjamin Russakoff, David Panisello Yagüe, Jolene Bowers, Talima Pearson

**Affiliations:** 1 Pathogen & Microbiome Institute, Northern Arizona University, Flagstaff, AZ USA; 2 Translational Genomics Research Institute, Flagstaff, AZ USA

**Keywords:** Microbial detection, Microbial quantification, Detection of *S. aureus*, Detection of MRSA, Xpert, ViPrimePLUS

## Abstract

**Background:**

Molecular assays are important tools for pathogen detection but need to be periodically re-evaluated with the discovery of additional genetic diversity that may cause assays to exclude target taxa or include non-target taxa. A single well-developed assay can find broad application across research, clinical, and industrial settings. Pathogen prevalence within a population is estimated using such assays and accurate results are critical for formulating effective public health policies and guiding future research. A variety of assays for the detection of *Staphylococcus aureus* are currently available. The utility of commercial assays for research is limited, given proprietary signatures and lack of transparent validation.

**Results:**

In silico testing of existing peer-reviewed assays show that most suffer from a lack of sensitivity and specificity. We found no assays that were specifically designed and validated for quantitative use. Here we present a qPCR assay, SaQuant, for the detection and quantification of *S. aureus* as might be collected on sampling swabs. Sensitivity and specificity of the assay was 95.6 and 99.9 %, respectively, with a limit of detection of between 3 and 5 genome equivalents and a limit of quantification of 8.27 genome equivalents. The presence of DNA from non-target species likely to be found in a swab sample, did not impact qualitative or quantitative abilities of the assay.

**Conclusions:**

This assay has the potential to serve as a valuable tool for the accurate detection and quantification of *S. aureus* collected from human body sites in order to better understand the dynamics of prevalence and transmission in community settings.

**Supplementary Information:**

The online version contains supplementary material available at 10.1186/s12866-021-02247-6.

## Background

*Staphylococcus aureus* is a Gram-positive bacterium that lives in close association with humans as both a commensal and pathogen. When these bacteria penetrate the outer layers of skin or mucosa, they can cause skin, soft-tissue, bone, joint, respiratory, and endovascular infections [1]. In the US, *S. aureus* caused almost 120,000 bloodstream infections and 20,000 associated deaths in a single year [2] and is typically the most common cause of skin and soft tissue infections [3].

Strict infection controls in hospital settings appear to be slowly reducing hospital acquired (HA) methicillin resistant *S. aureus* (MRSA) infection rates. In contrast, community acquired (CA) MRSA infections increased and have remained steady since 2008 [2]. In some settings, methicillin sensitive *S. aureus* (MSSA) infections outnumber MRSA infections 3 to 1, are increasing nationally, and therefore cannot be ignored [4, 5]. Indeed, MSSA carriage rates in the population are some 20-fold higher than MRSA carriage rates [6–8].

To better understand and mitigate *S. aureus* spread and infection, research has increasingly focused on identifying segments of the population likely to be carriers and thus at greater risk for autoinfection and transmission to others. Lower socioeconomic status has been linked to *S. aureus* infections and reflected in ethnic, race, and education disparities [9–11]. Colonization rates also differ among ethnic and racial groups [12, 13], however socioeconomic status does not appear to impact colonization status [12]. Longitudinal studies suggest that approximately 20 % of healthy individuals are persistently colonized, carry higher loads, and are at higher risk for developing infections [6, 7]. Sex and age have also been attributed to the likelihood of colonization [13, 14]. Our understanding of carriage in the population is almost completely based on culturing after swabbing. Quantitative assessments have mostly been restricted to culture-based enumeration which is time consuming and low-throughput. Furthermore, because growth in transport media often precludes quantitative assessments [15–17], approaches must be performed immediately after collection, unless the swab is stored at -70^o^C [18]. Recent work comparing 16 S rRNA gene sequencing to culturing, suggests that culture based methods are likely to lead to false negative results when absolute abundance of *S. aureus* is low [19]. The finding that females often have a lower absolute abundance than males has important implications for our understanding of the observed sex-based disparity in nasal carriage [7, 19–22]. Large-scale quantitative assessments may shed further light on defining persistent carriers and the risk of autoinfection and transmission if individuals with higher loads shed these bacteria more frequently and in greater abundance.

Quantitative PCR presents a rapid, high throughput, culture-independent, and highly-sensitive method for detection and quantification of *S. aureus* that can be performed directly on DNA extracted from collection swabs. A number of PCR assays have been developed for both research and commercial purposes, however commercial assays are typically difficult to evaluate as signatures are proprietary and published metrics are often neither transparent nor peer-reviewed. Other assays were not purposed for quantitative assessment or were evaluated using limited in silico data compared to the data available today [23–32]. Here we report on the design and evaluation of SaQuant, a novel qPCR assay intended for the accurate detection and quantification of *S. aureus* from sampling swabs.

## Results

### Assay efficiency

The assay yielded an average efficiency of 93.38 % across four separate standard curve serial dilution experiments, with an *r*^2^ > 0.999 in each case.

### Assay sensitivity and specificity

Assay sensitivity, the degree to which true positives are correctly detected, was calculated as 95.6 % from in silico comparisons which included exact matches to 1,738 of 1,818 *S. aureus* genomes (Table 1). Wet-bench assessment yielded a sensitivity value of 100 % and included amplification of 533 of 533 *S. aureus* isolates.

**Table 1:** In silico assessment of SaQuant and other *S. aureus*-specific assays

**Assay**	**S. aureus hits (*n*= 1818)**	**Other Staphylococci hits (*n*= 1834)**	**Sensitivity**	**Specificity**	**Reference**	**Forward**	**Reverse**	**Probe**	**Amplicon size**
SaQuant	1738	1	95.60	99.95	This one	AACTACTAGGGGAGCCTAATRAT	GGTACTAACCAAATCAGGTCATAA	TGGCTGAGATGAAYTGTTCAGACCC	73
1	898	1	49.39	99.95	[27]	GGCATATGTATGGCAATTGTTTC	CGTATTGCCCTTTCGAAACATT	ATTACTTATAGGGATGGCTATC	73
2	0	0	0	100	[23]	CAAAGCATCAAAAAGGTGTAGAGA	TTCAATTTTCTTTGCATTTTCTACCA	TTTTCGTAAATGCACTTGCTTCAGGACCA	Unknown
3	753	6	41.42	99.67	[28]	CAGCAAACCATGCAGATGCTA	CGCTAATGATAATCCACCAAATACA	TCAAGCATTACCAGAAAC	101
4	1780	11	97.91	99.40	[29]	AACTGTTGGCCACTATGAGT	CCAGCATTACCTGTAATCTCG		306
5	1499	0	82.45	100	[30]	GCGATTGATGGTGATACGGTT	AGCCAAGCCTTGACGAACTAAAGC	GGTGTAGAGAAATATGGTCCTGAAGCAAGTGCA	279
6^a^	1318	1	72.50	99.95	[31]	AATCTTTGTCGGTACACGATATTC	CGTAATGAGATTTCAGTAGATAATACAACA		108
7	117	0	6.44	100	[32]	AGTGAGCGACCATACAACAG	CATAATTCCCGTGACCATTT	AAGCACAAGGACCAATCGAGG	185
femB	1010	0	55.56	100	[33]	TTACAGAGTTAACTGTTACC	ATACAAATCCAGCACGCTCT		651

^a^Modified sequence to exclude the last 3 nucleotides as the published sequence is not predicted to amplify any *S. aureus* genomes

Assay specificity, the percentage of true negatives that are correctly excluded, included in silico and wet-bench assessments. In silico comparisons of 1,834 genomes from other species in the genus *Staphylococcus* suggest predicted amplification in only 1 genome (GCA_003185095.1_Staphylococcus_pseudintermedius_ST525_1), resulting in a specificity of 99.95 % (Tables 1 and 2). For this *S. pseudintermedius* genome, our assay predicted an amplicon on a single short contig of 339 nucleotides in length which also had a 100 % match to other *S. aureus* genomes. Reads were not available for this genome. We suspect that this genome is contaminated by *S. aureus*. Wet-bench testing of 10 strains from 7 non-*aureus Staphylococcus* species (Table 3) resulted in no amplification.

**Table 2:** In silico comparison of SaQuant to non - *S. aureus* genomes within the genus *Staphylococcus*

**Species**	**#Genomes**	**%Negative**
*S. lentus*	8	100
*S. fleurettii*	5	100
*S. vitulinus*	9	100
*S. felis*	22	100
*S. hyicus*	3	100
*S. agnetis*	16	100
*S. chromogenes*	59	100
*S. schleiferi*	9	100
*S. intermedius*	4	100
*S. delphini*	13	100
*S. pseudintermedius*	130	99
*S. massiliensis*	2	100
*S. carnosus*	5	100
*S. condimenti*	5	100
*S. simulans*	49	100
*S. argensis*	3	100
*S. pettenkoferi*	14	100
*S. auricularis*	4	100
*S. kloosii*	3	100
*S. arlettae*	23	100
*S. nepalensis*	8	100
*S. cohnii*	50	100
*S. succinus*	21	100
*S. equorum*	44	100
*S. xylosus*	53	100
*S. gallinarum*	17	100
*S. saprophyticus*	88	100
*S. hominis*	113	100
*S. epidermidis*	530	100
*S. lugdunensis*	13	100
*S. caprae*	11	100
*S. capitis*	48	100
*S. psasteuri*	23	100
*S. warneri*	57	100
*S. devriesei*	8	100
*S. petrasii*	5	100
*S. haemolyticus*	219	100
*S. schweitzeri*	6	100
*S. argenteus*	20	100
Other^a^	114	100

^a^Genomes did not cluster with other members with the same species designation, suggesting an unreliable species designation

**Table 3:** Species used for wet-bench testing of SaQuant specificity

**Species**	**No. Strains**	**Assay Amplification**
*S. captis*	1	none
*S. epidermidis*	4	none
*S. haemolyticus*	1	none
*S. hominis*	1	none
*S. intermedius*	1	none
*S. saprophyticus*	1	none
*S. warneri*	1	none

### Assay limit of detection and limit of quantification

At concentrations of three and five genomic equivalents, respectively 17/20 (85 %) and 19/20 (95 %) replicates were detected. Thus, the assay’s LoD lies between 3 and 5 copies, or approximately 9.07 × 10^− 5^ ng and 1.51 × 10^− 4^ ng. Lower *S. aureus* amounts can still be detected, albeit at less than the desired confidence level of 95 %. Given our parameters, the assay recorded an LoQ of 2.5 × 10^− 5^ ng, or approximately 8.27 genomic equivalents, with a Ct value standard deviation of 0.73 across 8 dilution replicates.

### *S. aureus* detection in complex community samples

All nine community samples were *S. aureus* culture-negative and confirmed by 16 S rRNA sequencing to contain bacterial DNA. The DNA load for each of the samples was quantified using an Invitrogen Qubit 4 fluorometer and ranged from 1.24 to 21.9 ng/µl for samples from the throat and nose. DNA loads on the three samples collected from hands (palm) were too low to detect (Table S1). Each complex sample and *S. aureus* template combination was assayed in triplicate. For all but a single sample, *S. aureus* was detectable at the level of 1 GE (at least one replicate amplified). At this concentration, only one community sample amplified in all three of the replicates; 5 samples amplified in only one replicate while 2 amplified in two replicates. At the amounts of 100 and 10,000 GEs, every sample yielded a quantifiable result. Overall, quantification of the spiked *S. aureus* DNA via SaQuant was very similar to the amount spiked into each sample (Table S1).

The variation of quantification values within triplicate sets decreased with the amount of spiked DNA. Absolute quantification values are indicated in Table S1. Control samples (with no added *S. aureus* DNA) did not amplify.

## Discussion

In silico testing of this assay included all publicly available genome assemblies of *S. aureus* and other *Staphylococcus* species and represent the most comprehensive testing of any *S. aureus* assay to date. In silico comparisons of sensitivity and specificity to previously published assays suggest that our assay is likely to perform better than others. The SYBR Green assay (Assay #4 in Table 1) published by Paule et al. [29], has a slightly higher predicted sensitivity, but lacks a probe, making specificity a concern, and the amplicon size is larger than optimal. Our assessment excluded commercial assays that incorporate proprietary genomic signatures. While quantification may be possible with other assays, our assay was developed and assessed specifically to address the need for research studies that quantify the colonizing load of *S. aureus* in community-based population studies of prevalence and transmission. Wet-bench assessment of this assay indicates an LoD of 3–5 copies (near the theoretical limit for qPCR detection), and an LoQ of ~ 8 copies. In silico and wet bench testing focused on determining the potential for false positive amplification of closely related species, especially those that may be prevalent in human swab samples. These non-*aureus* samples were tested at template amounts of 1 ng per reaction, a concentration that is considerably higher than expected on a swab sample, providing a rigorous screening of possible false positive amplification. Wet-bench application of the assay to swabs from three body sites evidenced the assay’s specificity in the context of complex communities. Furthermore, these experiments demonstrate the assay’s resilience to PCR inhibition that can be caused when the amount of DNA in a reaction is high. Our results suggest that in a complex sample as might be found on a swab, this assay excels at accurately detecting and quantifying low levels of *S. aureus*, and discriminating the target species from other common and related species. Although we have envisioned the assay’s application primarily in the research setting, we recognize its potential usefulness elsewhere, e.g. as a clinical detection method, or in food quality control where cell viability is not guaranteed.

## Conclusions

Quantity of *S. aureus* colonizing human body sites is likely to be central to the probability of transmission and auto-infection. An accurate and high-throughput means of quantifying *S. aureus* directly from human samples without culturing will provide an important tool to better understand the epidemiology of this pathogen. To this end, we present a quantitative PCR assay with high sensitivity and specificity and validated in complex microbial samples such as those found in the human nares and throat.

## Methods

### In silico assay design

All genome assemblies annotated as *Staphylococcus* (*n* = 17,883) were downloaded from Genbank on July 6, 2020 with the ncbi-genome-download tool (https://github.com/kblin/ncbi-genome-download). Duplicate genomes were removed with the assembly-dereplicator tool (https://github.com/rrwick/Assembly-Dereplicator) at a similarity threshold of 99.9 %, resulting in a set of 3,652 unique genomes. De-replicated genome assemblies were aligned against *S. aureus* NCTC9752 (LS483310) with NUCmer v3.1 [34] in conjunction with NASP v1.2.0 [35]. A phylogeny was inferred on a concatenated alignment of 7,492 SNPs with FastTree v2.1.8 [36]. The phylogeny was used to speciate *S. aureus* from other species. Of these 3,652 genomes annotated as *Staphylococcus*, 1,818 were *S. aureus*.

The core genome of *S. aureus* was calculated with the large-scale Blast score ratio (LS-BSR) tool [37] on a set of 1,818 genomes, using cd-hit v4.8.1 [38] for clustering and blat v36 × 2 [39] for alignment. The core genome consists of those coding region sequences (CDSs) that have a blast score ratio (BSR) value [40] of > 0.8 across all *S. aureus* genomes. The core genome (*n* = 4,892 CDSs) was then aligned against all *Staphylococcus* genomes with LS-BSR and CDSs unique to *S. aureus* were identified; these CDSs had a BSR value ≤ 0.4 in all non-target genomes. PCR primers were designed for unique signatures with Primer3 v2.3.6 (PMID: 22,730,293). The primers were then visually compared to multiple sequence alignments of the target CDS with JalView [41]. Degeneracies were added to the primer forward primer and probe sequences to capture diversity across *S. aureus*.

The assay employs a forward primer (5’ AACTACTAGGGGAGCCTAATRAT 3’), a reverse primer (5’ GGTACTAACCAAATCAGGTCATAA 3’) and a TaqMan TAMRA probe (5’ TGGCTGAGATGAAYTGTTCAGACCC 3’). The assay has an amplicon size of 73 base pairs, and targets a region within a 132 bp long hypothetical protein coding sequence (NCBI Protein accession number: YP_500811.1).

### Culturing and DNA extraction

Community [42] and clinical samples of *S. aureus* stored in glycerol stocks were thawed, streaked for isolation onto CHROMagar media, and incubated for 24 h at 37 °C. Colonies appearing pink to mauve were selected and cultured for another 24 h at 37 °C on CHROMagar before DNA extraction. To determine assay specificity, glycerol stocks of seven different species of *Staphylococcus* were cultured on blood agar (TSA + 5 % sheep blood) and grown at 37 °C for 24–26 h. Isolated colonies were added to an enzymatic lysis buffer before final extraction. DNA was extracted using the Qiagen DNEasy Blood and Tissue Extraction kit. Extraction quantity was determined using an Invitrogen Qubit 4 fluorometer.

### Assay conditions

We independently varied primer annealing temperature, and primer and probe concentrations to optimize assay conditions for increased efficiency and reduced the likelihood of false amplification. After optimization, real time qPCR was run in 10 µl reactions using 5 µl of Applied Biosystems TaqMan Universal PCR MasterMix, 1 µM of forward primer, 1 µM of reverse primer, 200 nM of TaqMan TAMRA probe, and 1 µl of template. Thermal cycling conditions were as follows: hot start TaqMan activation (10 min at 95 °C), followed by 40 cycles of denaturation (15 s at 95 °C) and extension (1 min at 57 °C).

### Assay validation

We measured sensitivity by calculating the percentage of *S. aureus* isolates or genomes that were correctly identified by the assay. Wet-bench determination of sensitivity included testing against 533 *S. aureus* isolates collected in Yuma, Arizona [42] and sequenced to verify species designation. In silico assessment of sensitivity for this and other *S. aureus* assays involved an in silico PCR screen against 1,818 *S. aureus* genomes (Table 1) using vipr (https://github.com/TGenNorth/vipr). A hit was recorded for exact matches to both primers and the probe. To determine assay specificity, we calculated the percentage of non - *S. aureus* isolates or genomes that were correctly identified as such by the assay. In silico assessment of specificity was also performed using vipr against 1,834 genomes that were annotated as *Staphylococcus*, but are not *S. aureus* (Tables 1 and 2). Wet-bench assessment included 10 strains from 7 *Staphylococcus* species that were not *S. aureus* (Table 3).

We determined assay efficiency, the extent to which the template DNA is doubled every cycle, by averaging the efficiency values from four separate experiments, each with a standard curve constructed from five serial dilutions. We defined the limit of detection (LoD) as the least amount of analyte that amplified at least 95 % of the time and approximated this value by determining the amount of DNA present when 19 of 20 replicates amplified at less than 40 cycles, with uniform curve morphology, and with no amplification of the negative control [43]. The limit of quantification (LoQ) of this assay is the smallest amount of analyte that can be detected and quantified with an acceptable probability and level of accuracy. Here, we defined LoQ as the least amount of analyte that amplified 8 out of 8 times, and whose amplification curves had a cycle threshold (Ct) value standard deviation (σ) of less than 0.8.

We conducted proof-of-principle experiments to emulate *S. aureus* detection from complex bacterial communities by spiking *S. aureus* DNA into samples collected via swabs from different body sites. A total of nine complex samples were used, consisting of three human nares swabs, three human throat swabs, and three human palm swabs. These swabs were all paired with a swab collected from the same body site from the same person, but were used for culture-based detection of *S. aureus*. The swab samples representing complex samples were chosen for qPCR detection of *S. aureus* because their pair was culture-negative suggesting that only spiked *S. aureus* DNA would be present. DNA from these swabs was extracted with a Qiagen DNEasy Blood and Tissue Extraction kit and quantified with an Invitrogen Qubit 4 fluorometer. These nine community samples were each divided into twelve aliquots: three no spike controls, three aliquots into which 1 genome equivalent (GE) of template was added, three aliquots into which 100 GEs of template were added, and three aliquots into which 10,000 GEs of template were added. Each replicate was assayed, and amplification was quantified via standard curve analysis, using the Applied Biosystems QuantStudio 12 software package.

## Supplementary Information

**Additional file 1.**

## Data Availability

All data analyzed during this study include all genome assemblies annotated as *Staphylococcus* (n = 17,883) that were downloaded from Genbank (https://www.ncbi.nlm.nih.gov/genome/?term=staphylococcus) on July 6, 2020. Data generated in this study are included in this published article and its supplementary information file.

## Abbreviations

HA: Hospital acquired
MRSA: Methicillin resistant *S. aureus*
CA: Community acquired
MSSA: Methicillin sensitive *S. aureus*
LoD: Limit of detection
LoQ: Limit of quantification
Ct: Cycle threshold
GE: Genome equivalent
